# Bovine Vaccinia: Insights into the Disease in Cattle

**DOI:** 10.3390/v10030120

**Published:** 2018-03-09

**Authors:** Ana Carolina Diniz Matos, Izabelle Silva Rehfeld, Maria Isabel Maldonado Coelho Guedes, Zélia Inês Portela Lobato

**Affiliations:** Laboratório de Pesquisa em Virologia Animal, Departamento de Medicina Veterinária Preventiva, Escola de Veterinária, Universidade Federal de Minas Gerais, Belo Horizonte, Minas Gerais 31270-901, Brazil; matos.acd@gmail.com (A.C.D.M.); izabellerehfeld@yahoo.com.br (I.S.R.)

**Keywords:** zoonosis, *Vaccinia virus*, bovine vaccinia, orthopoxvirus, veterinary, cattle, public health

## Abstract

Bovine vaccinia (BV), caused by *Vaccinia virus* (VACV), is a zoonosis characterized by exanthematous lesions in the teats of dairy cows and the hands of milkers and is an important public health issue. Severe VACV-induced lesions in the teats and udder of cows and buffaloes could lead to mastitis and other secondary infections, thereby reducing productivity and resulting in economic losses to the dairy industry. In Brazil, BV re-emerged in the late 1990s and is now endemic in most of the Brazilian territory. In the last 15 years, much effort has been made to know more about this disease and its epidemiology, etiologic agents, and interactions with the host and the environment. In this review, we describe the known dynamics of VACV infection in cattle and the viral shedding routes, as well as the relevance of BV for animal and public health.

## 1. Introduction

Poxviruses infect many invertebrate and vertebrate species, causing diseases that are of great veterinary and public health concern. The genus *Orthopoxvirus* includes at least 10 antigenically related species with a wide geographical distribution and variable spectra of vertebrate hosts [1,2]. With the exception of *Variola virus* (VARV), the smallpox etiologic agent, which is a strictly human pathogen, orthopoxviruses that are pathogenic to humans and animals include *Cowpox virus* (CPXV), *Monkeypox virus* (MPXV), and *Vaccinia virus* (VACV) [2].

Smallpox was a devastating disease, responsible for hundreds of millions of cases worldwide, with a mortality rate of one-fifth or more of infected people, until the middle of the twentieth century [3,4]. Highly effective cross-protection among orthopoxviruses enabled the use of CPXV and, later, VACV in the 19th and 20th centuries to prevent smallpox infection, leading to the term “vaccination” [3]. VACV has had an important role in human history owing to its highly effective use as an immunizing agent in the smallpox vaccination campaign, resulting in the global eradication of this deadly disease in 1980 [3].

After the cessation of VACV vaccination, the human population without immunity against smallpox and all other zoonotic orthopoxvirus infections has increased, and zoonotic orthopoxviruses have emerged worldwide [5]. Notable examples include the emergence of CPXV in Europe, MPXV in many African countries, and VACV in India and Brazil [6,7].

Buffalopox is an emerging contagious zoonosis associated with sporadic outbreaks in Asian buffalo (*Bubalus bubalis*) herds in India, Egypt, Pakistan, Nepal, Bangladesh, and Italy [8,9]. A phylogenetic analysis based on three genes confirmed that the buffalopox virus is closely related to VACV and it was taxonomically identified as a VACV strain [8]. Additionally, on the basis of sequence and phylogenetic analyses of the A56R gene, isolates from cows presenting poxvirus-compatible lesions in 2002–2006 throughout India were more closely related to VACV strains than to CPXV [10].

In Brazil, VACV was initially studied in the 1960s during a Brazilian government effort to survey rural regions for virus circulation, when the first Brazilian VACV was isolated from a wild rodent (*Oryzomys* genus) captured in the Brazilian Amazon basin [11]. From that date, or even previously, the occurrence of exanthematous zoonotic disease affecting humans and dairy cows was reported, although in a sporadic manner, and the etiological diagnosis was generally not performed, resulting in a lack of important epidemiological information about VACV circulation in the country [12,13]. However, since the late 1990s, reports of an exanthematous disease affecting cattle and humans have increased [12,13,14,15,16,17,18,19,20,21,22,23,24,25] and have reached endemic proportions in many regions of Brazil. The VACV outbreaks in Brazil are associated with dairy cows and the dairy workers who have direct contact with sick cows. This zoonotic disease was named bovine vaccinia (BV).

There are controversies about the origins of the Brazilian VACV strains. One hypothesis is that these strains originated as an independent, distinct lineage of New World Orthopoxviruses [26,27]. In contrast, there is the hypothesis that the Brazilian VACV strains are derived from the vaccine strain IOC, which was widely used during the smallpox eradication vaccination campaign in Brazil [28,29]. In this last case, it is proposed that the Brazilian VACV strains are derived from an “escaped vaccine strain” originated from an ancient vaccine strain related to the horsepox virus, that established an epidemiological cycle in domestic and/or wild animals after its escape to nature [28,29]. So far, the available data suggest that there is circulation of two different Brazilian VACV lineages, which probably have a distinct evolutionary history [26,27,28,29].

These two genetically distinct groups (Group 1 and Group 2) [26,30] have shown differences in pathogenesis and virulence when inoculated in mice and/or rabbits [25,31,32,33,34]. Mouse and rabbit VACV infection models have demonstrated variation in pathogenesis and virulence among strains as well as a systemic infection in which viral DNA could be detected in urine, feces, saliva, and nasal secretions [25,31,32,33,34]. Infections caused by VACV strains belonging to Group 1 do not cause systemic clinical signs in infected mice, whereas the strains belonging to Group 2 cause clinical signs that may lead to death [25,31]. Despite the existence of both groups in Brazil, Group 1 viruses are isolated more frequently than Group 2 viruses. In particular, 92% of the isolated clones are classified as Group 1, whereas only 8% belong to Group 2 on the basis of an analysis of the A56R gene [25].

Among other South American countries, cutaneous lesions associated with VACV infection have only been described in dairy farmworkers in Colombia [35]. However, VACV circulation in cattle has been detected by serological and molecular diagnosis in Argentina [36] and Uruguay [37], although no clinical signs related to VACV infections have been reported in cattle or in humans in these countries.

In addition to the public health impact, it is also important to emphasize that infectious animal diseases are estimated to be responsible for about 20% of losses in animal production worldwide [38]. According to the Food and Agriculture Organization of the United Nations (FAO), the global demand for animal proteins (i.e., milk, eggs, and meat) is expected to increase by 70% by 2050 [38]. In cattle and buffalo herds, VACV infections are characterized by severe local lesions affecting the udder and teats of lactating animals, leading to mastitis and other secondary infections in more than 40% of affected animals [13]. These infections reduce the productivity of milk by 40–80% and impact milk and cheese producers, mainly the small ones, and the dairy industry [6,13]. Additionally, in farms in which suckling calves are in direct contact with the cows, it is common to observe sick calves presenting lesions in the mouth, which reduce food intake, leading to weight loss [13,18].

BV in Brazil re-emerged in the late 1990s and is currently endemic in most of the Brazilian territory. In this review, we describe VACV infection in cattle, the known viral shedding routes, and the importance of BV for animal and public health.

## 2. Bovine Vaccinia Pathogenesis: Evidence of a Systemic and Persistent Infection

BV is a zoonosis caused by VACV and is associated with rural environments. The most affected population includes farmers and rural workers who have direct contact with infected cattle. Nodular, ulcerated, necrotic, and painful lesions are observed mainly on the hands and arms of infected people following contact with infected animals during the milking process [13,39,40,41]. Human-to-human transmission has been suggested to have occurred in some BV outbreaks in Brazil [42], such as infection in indoor environments [43].

Characteristic lesions of poxvirus have been observed in all BV outbreaks in Brazil. In humans, in addition to the typical lesions found on the hands, fingers, and arms, lesions on the face have been described [15,39,44]. Systemic clinical signs are frequently observed during the clinical course of BV in humans, such as myalgia, headache, anorexia, arthralgia, and lymphadenopathy [13,39,40,41,44,45].

In cattle herds, during BV outbreaks, lactating cows are the most frequently affected category, presenting multiple lesions located on the teats and sometimes the udder. In farms with suckling calves that are in direct contact with the cows, it is common to observe lesions on the nuzzles, lips, and oral mucosae of the offspring [13,18,20]. In cattle, the BV clinical course is characterized by a short incubation period (2–3 days) and the appearance of a maculopapular rash that progresses to papules, vesicles, pustules, and subsequently to scab lesions, which heal about 20 days after infection [13,46,47,48].

Another concern for BV in Brazilian dairy cattle herds is the rapid spread of the disease in the herd. In affected farms, a high attack rate has been observed, which can reach up to 100% of the lactating cows and calves in a herd [13,20]. A few measures for the clinical recovery of the animals can be taken, such as lesion disinfection, to prevent secondary infections [13,20].

Despite studies of Brazilian VACV strains in mice, rabbits, and cell cultures, little is known about the pathogenesis of VACV in cattle. Poxvirus lesions were thought to be limited to the site of infection, and the disease was thought to be acute and self-limited [48]. To elucidate the clinical and histological aspects and better understand the pathogenesis of BV, Guarani P2 (GP2), a Group 1 Brazilian VACV strain, was experimentally inoculated intradermally in the teats of cows. A clinical course and other disease characteristics were observed from the day of inoculation until the complete healing of the lesions [46,47,49]. BV was successfully reproduced with the development of localized lesions at the site of infection [46,47,49]. The monitoring of bovine vital parameters indicated clinical manifestations in addition to localized lesions in the site of inoculation, including increased retro mammary lymph nodes and mastitis. Fever was not detected [46]. Leukogram analyses of the infected cows indicated reactive lymphocytes in small concentrations, as well as lymphocytosis, which may suggest a response to viral infection [46]. Neutrophilia has also been observed and may be associated with secondary infections, such as mastitis [46]. At necropsy, ulcerative dermatitis on the teats, mastitis, and hyperplasia of the retromammary lymph nodes were the main gross alterations observed [46]. The histopathological findings in skin samples agreed with the progression of clinical signs, including acute ulcerative lesions 4 days post-infection (DPI) and typical features of chronicity, with a trend toward healing at 9 DPI [50].

**Figure 1:**
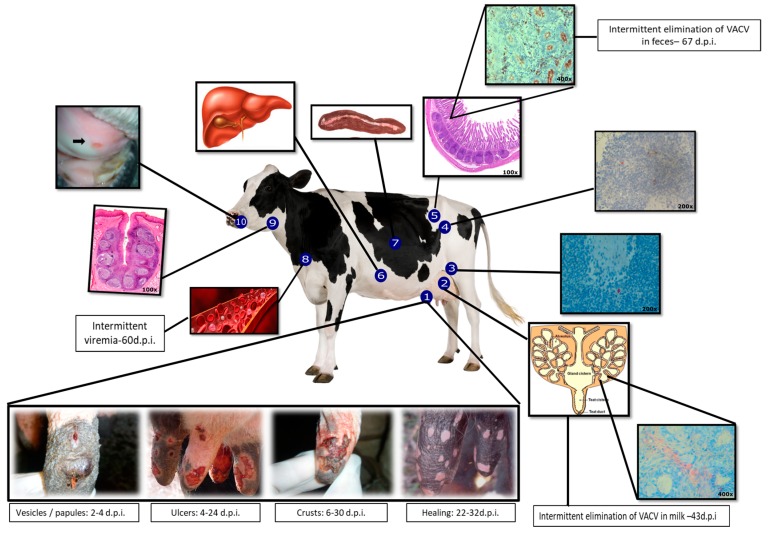
Clinical signs and the proposed model of *Vaccinia virus*-Guarani P2 (VACV-GP2) pathogenesis in cattle. Multiplication of VACV in the teat epithelium and dissemination by lymphatic and hematogenous routes. 1. Teats; evolution of lesions: primary inoculation site [46,48]; 2. Mammary gland [50,52]; 3. Retromammary lymph node [49]; 4. Mesenteric lymph node [49]; 5. Ileum [49]; 6. Spleen [49]; 7. Liver [49]; 8. Intermittent viremia [47,49]; 9. Tonsil [50]; 10. Ulcer in the oral mucosa [46].

Evidence for prolonged and intermittent viremia was found in the blood of experimentally infected dry cows that were monitored for 36 DPI [47] and in lactating cows for up to 67 DPI [49] (Figure 1). Furthermore, other studies by our group have shown the presence of VACV DNA and infectious virus particles in the milk of naturally [51] and experimentally infected cows [52] (Figure 1). VACV detection in milk from cows experimentally infected directly from the mammary gland without contact with the lesions and scabs present on the teat epithelium, even from non-infected teats and collected by the introduction of a catheter into the mammary ostium, suggests that the presence of VACV in milk may be associated with a systemic viral infection due to viremia [52].

Another interesting finding in experimentally infected cows was the detection of VACV DNA in the feces of 50% (4/8) of the animals at the first DPI and up until 67 DPI [49]. Immunolabeling in mesenteric lymph nodes and ileum was observed in all animals in macrophages and lymphocytes and in the goblet cells (ileum) of the animals at necropsy, which occurred at 92 DPI [49] (Figure 1). These data indicate that the gut cells may be infected very early on, and that the infection may persist, even after the complete healing of the teat lesions.

Histopathological and immunohistochemical (IHC) analyses of several tissues, such as the spleen, liver, and tonsils, from VACV-inoculated cows identified more evidence of VACV systemic infection in cattle [49,50] and suggested that the virus does not remain at the site of inoculation/infection. VACV was detected in macrophages in the perivascular region of the dermis, indicating that the virus may spread to other tissues through lymphatic and/or blood vessels [50]. Virus immunolabeling in other tissues, especially in the retromammary lymph nodes, was observed from the acute phase of infection (4, 9, and 17 DPI) until the complete healing of the lesions (80 and 180 DPI) [50] (Figure 1).

On the basis of observations of the occurrence of the disease in the field and of studies of experimental infections, it was proposed that intradermal VACV infection in cattle teats starts with the virus penetration into the local epithelium through a previous wound or even a microscopic breakage of the skin barrier, where primary viral multiplication occurs with formation of vesicular and exanthematous lesions (papules, vesicles, and ulcers). After replication at the entry site and with penetration into the dermis, the viral particles can spread rapidly through the blood and lymphatic vessels, reaching the regional lymph nodes (mainly retromammary) and spreading to the mesenteric lymph nodes and ileum lymphoid tissue (Peyer’s plaques), epithelial, and goblet cells. From there, the virus would then be excreted in the feces. In parallel, the dissemination of VACV would occur through the blood and lymphatic pathway to other lymphoid tissues, such as the spleen, liver, tonsils, and other lymph nodes (Figure 1).

In contrast to the previously described progression of VACV infection, characterized by a localized, acute, and self-limited disease [13,48], in experimentally infected and immunosuppressed cattle, ulcerative lesions in the oral mucosa [46] (Figure 1), reactivation of viremia, and viral excretion through the feces suggest that viral reactivation occurs in immunosuppressed animals, even in the presence of neutralizing antibodies [49].

The immune response to poxvirus infections usually leads to protection via antibody production, which controls the infection by various mechanisms, such as virus neutralization, complement system activation, cytotoxicity, and opsonization. The resolution of the infection is associated with the activation of specific CD8^+^ lymphocytes that attack the remaining infected cells [53].

After VACV infection, a strong antibody response is generated. In mice, low levels of antibodies are detected until 7 DPI. Then, high levels of IgM and multiple IgG isotypes are present from 14 DPI [54]. Neutralizing antibodies are identified at 20 DPI and persist for longer than three months [55,56].

Our group has studied the dynamics of VACV infection and the subsequent humoral response in cattle. In serological studies of naturally infected dairy cows and their suckling calves, peak levels of IgG and neutralizing antibodies were detected within the first month of infection, and, even at lower titers, they were detectable for up to one year after the occurrence of outbreaks in the farms [57].

In lactating cows, after experimental infection with the VACV strain GP2, an antibody response could be detected at 10 DPI, and by 16 DPI. IgG2 and neutralizing antibodies could also be detected, and their levels peaked at 40 DPI. IgG1 antibodies were detected at higher levels than IgG2 and persisted at high levels until 20 DPI [57]. Both B cell and CD4^+^ lymphocyte activation was significantly elevated post-infection, particularly at 30 DPI. The frequencies of the T cell memory populations CD45R0^+^CD3^+^CD4^+^ and CD45R0^+^CD3^+^CD8^+^ were higher at 30 DPI than on the day of infection [58].

To address the possibility of cows having had the clinical disease becoming ill again, we experimentally infected and re-infected the cows 70 days after the first infection [46]. Although they already had circulating neutralizing antibodies against BV, they developed teat lesions, which were milder when compared to the ones developed after the primary infection and with a shorter clinical course, with complete healing at 10 DPI compared to 22–32 DPI for the first inoculation [46]. In addition, two cows taken 240 days after initial infection, which did not have detectable antibody titers, were re-infected and subsequently presented lesions that were more severe than those of the cows that were re-infected and had circulating antibodies [46]. These findings indicate that anti-VACV antibodies in cattle may not completely protect against the disease but may minimize its clinical manifestation.

## 3. Bovine Vaccinia: Viral Shedding Routes and Insights into Its Epidemiology

Outbreaks of BV mainly affect small dairy properties, characterized by hand-milked herds with cross-bred animals and presenting poor sanitary and management infrastructure [13,20,59], but the disease has also been described in properties with mechanical milking process. In general, lactating cows and their calves manifest the clinical signs of the disease and are the most affected categories in the herds [13,20,59].

**Figure 2:**
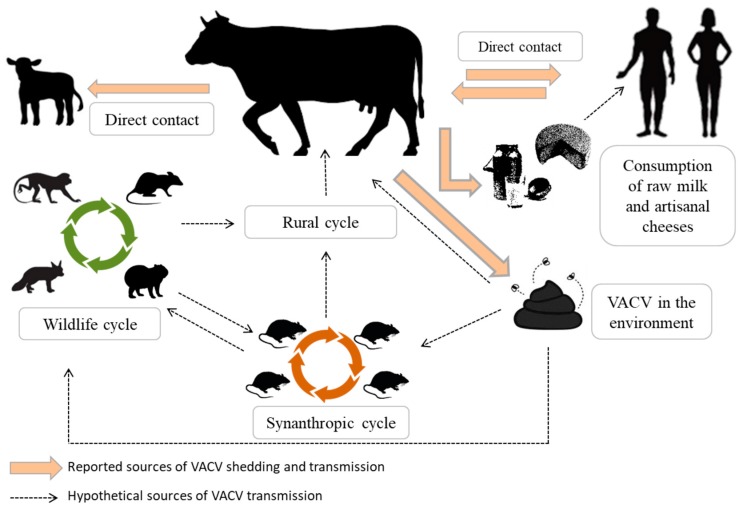
Reported and hypothetical sources of VACV transmission from a cow affected by bovine vaccinia (BV). Lesions are a source of VACV transmission by direct contact, and the virus could be eliminated via feces and milk. Bovine feces contaminated with VACV may be responsible for viral maintenance in the environment. Contaminated milk may be a source of VACV transmission via consumption, also to humans.

The main route of VACV transmission among cows is by the hands of milkers (Figure 2) or the suction cups of the milking equipment [13]. In Brazil, despite the high level of milk production, hand milking is still used extensively, which is a risk factor for BV occurrence and transmission [13]. Between farms, the disease could be transmitted by the introduction of infected cattle into the herd or even by milkers who come in contact with sick animals in other farms [13,17]. In northern Brazil, animal trading and movement were shown to be important for the spread of BV into different regions [60]. Studies using experimental animal models, such as mouse and rabbit models, have shown that VACV infection can occur by other routes, such as the nasal and oral routes [31,33,34,61,62,63]. Another possible source of VACV dissemination within and among cattle herds in rural environments could be through direct or indirect contact with other VACV-infected cattle, with other animal species, including humans, and even with secretions and excretions, such as milk and feces, respectively (Figure 2). The detection of viremia in asymptomatic dry cows and bulls and in animals from neighboring farms without reports of BV outbreaks supports the potential presence of other VACV infection routes in cattle [59].

Viral circulation in wildlife and in peridomestic rodents has been demonstrated and could be related to viral maintenance in nature or even to viral dissemination between farms [64,65,66] (Figure 2). Moreover, VACV has been detected in blood samples from monkeys [67], dogs, opossums [68], cats from urban environment [69], various small rodents and marsupials [70], and even from the largest species of rodents in the world, capybaras [71]. Despite evidence for VACV circulation in several wild and domestic animal species, VACV reservoirs and the role of wildlife in BV outbreaks remain unclear. In addition, viremia and the high prevalence of seropositive cattle in farms without reports of the disease suggest that VACV circulation within and among farms can occur even without the occurrence of the clinical disease [20,59].

VACV shedding through feces has been demonstrated in cows experimentally infected with the VACV-GP2 strain [47,49]. Moreover, viable VACV has been detected in the feces of cattle with and without BV clinical signs [59]. The horizontal transmission of VACV through feces was evaluated by exposing sentinel mice to wood shavings contaminated with bovine feces [62]. VACV DNA was detected in the feces of the sentinel mice, demonstrating the infectivity of the virus particles eliminated in bovine feces and a potential source of viral circulation among cattle and rodents [62]. The presence of VACV viable particles in the feces in the environment is a potential continuous source of infection. It has been shown that VACV particles could remain viable in protein-rich media for up to 8 months [72]. Furthermore, the persistence of VACV infectious particles in the feces from infected mice has been demonstrated for up to 20 days post-exposure [61]. Fecal shedding could be important for viral maintenance in the environment and a source of viral transmission among species, including humans (Figure 2).

VACV is sensitive to disinfectants such as sodium hypochlorite [73]. De Oliveira et al. (2011) [73] studied the susceptibility of *Vaccinia virus* to chemical disinfectants and showed that hypochlorite and quaternary ammonium combined with chlorhexidine or glutaraldehyde could be used as recommended products for the control of BV. Sodium hypochlorite is the most inexpensive, but to be effective, it is recommended to be stored in opaque containers, protected from sunlight, and to be discarded whenever contamination with organic matter occurs. Field personnel could implement the use of sodium hypochlorite at 0.5% (*v*/*v*) to disinfect gloves before and after hand milking, such as the use of iodine and hypochlorite for pre- and post-milking teat disinfection, also known as pre- and post-dipping processes, followed by rinsing. Thereby, this disinfection protocol would be important to control the disease and to decrease the viral circulation in the herd, reducing the possibility of infection/re-infection in some farms. A case-control study of properties with and without cases of BV reinforced the idea that the use of disinfectants in dairy farms is directly related to protection against BV [20].

Although different potential VACV reservoirs have been identified in wild and peridomestic environments, cattle are the main source of VACV transmission to humans. It is known that the control of zoonotic pathogens at their animal source is the most effective and economic strategy for protecting people [30]. Given the numerous cases of BV reported in recent years in Brazil and its wide occurrence in the country, the development of a safe and effective vaccine to be used in cattle has become an emerging demand. To address the need for an efficient BV preventive measure with the aim of mitigating BV outcome in animals and humans, we developed and tested different vaccine formulations against VACV in a murine model [74]. The use of the VACV Brazilian strain GP2 as the antigen, formulated with aluminum hydroxy- and saponin-based adjuvants, resulted in higher neutralizing antibody production and disease protection in mice when compared to the other tested vaccine formulations [74]. Furthermore, the efficacy of this vaccine was tested in cattle [75]. The induction of neutralizing antibodies and an early activation of CD4^+^ and CD8^+^ lymphocyte responses after the challenge, conferring protection against the clinical manifestation of the disease in vaccinated heifers, revealed that this vaccine is a promising tool to control and prevent BV in cattle herds in Brazil [75].

## 4. *Vaccinia virus*: A Possible Foodborne Pathogen

The detection of VACV DNA and infectious particles in milk samples collected from cows in BV outbreaks, associated with the intermittent secretion of VACV in milk observed in cows experimentally infected with VACV, calls attention to the potential public health risk associated with the consumption of raw milk and/or artisanal cheeses, which are produced with raw milk, during BV outbreaks [51,52] (Figure 2). VACV DNA in milk was detected until 67 DPI, after teat lesions healed, in experimentally infected lactating cows [52]. The risks associated with the ingestion of contaminated milk were demonstrated in a murine model by virus immunolabeling in lung, kidney, spleen, ileum, liver, submandibular lymph node, and bladder, indicating a systemic infection in the absence of clinical signs [63].

Studies of experimentally contaminated milk and artisanal cheeses produced with this milk have demonstrated the viability of VACV after a heat treatment at 65 °C, as well as in artisanal cheese and milk stored at low temperatures [76]. Moreover, viable VACV has been detected in artisanal cheese samples produced with raw milk from experimentally infected dairy cows [77]. Considering that the virus is secreted via the milk and that it remains viable during the cheese production process, our group analyzed the impact of the ripening process on the viability of VACV in artisanal cheeses [78]. Despite a decrease in the viral load in cheeses over time, VACV remained viable for up to 60 days of ripening [78]. Additionally, our group detected VACV in commercial artisanal cheeses produced with raw milk from properties with (8/10) and without (35/49) BV outbreaks [79]. Moreover, infectious viral particles were recovered from 11 samples at different ripening times, among which four cheeses were from properties without a history of BV outbreaks [79].

The actual prevalence of VACV-contaminated milk derived from infected cows and the real risk of infection from consuming contaminated milk and dairy products are still unknown. To date, there are only two reports of exanthematous oral lesions in humans associated with the consumption of milk supposedly contaminated with the buffalopox virus [8,80]. Despite the impact of BV, epidemiological surveillance is not sufficient to control the disease, and the number of cases is probably underestimated. Viral particles have already been detected in the milk of naturally infected animals; therefore, the consumption of milk and products contaminated with VACV is possible (Figure 2). In Brazil, the seroprevalence of orthopoxvirus (OPXV) and the potential risk factors related to VACV infections were determined in humans living in regions endemic for BV [81]. Interestingly, anti-OPXV neutralizing antibodies were detected in humans, including children in the same family, who never had the disease nor contact with diseased cattle. However, many of them often consumed unpasteurized milk or artisanal cheeses [81]. Although a causal relationship was not established in the study, the results suggest that the consumption of VACV-contaminated milk and dairy products is a possible source of viral infections [81]. Mice fed VACV-contaminated milk did not show any clinical signs but exhibited a systemic infection, as evidenced by viral detection by polymerase chain reaction (PCR) and immunohistochemistry (IHC) in various tissues, and eliminated VACV intermittently via feces and saliva [63].

The raw milk used for the production of cheeses and dairy products has the potential for disease dissemination, either from microorganisms shed in milk and/or from those acquired from the use of contaminated utensils at the time of milking. However, studies of viral infections caused by contaminated dairy products are rare in comparison to studies of foodborne outbreaks of bacterial origin. Although the known natural routes of VACV infection do not include the ingestion of food containing the virus, milk can be contaminated with viable viral particles, as crust and secretions of the lesions present in the teats and udders can be released during milking, thereby contaminating the milk. In BV outbreaks, the use of raw milk for cheese production may pose a risk to the cheesemaker, who normally handles the dough without any protection (such as gloves), or to people who consume these products, posing a public health risk (Figure 2).

The growing consumption of dairy and other livestock products provides important nutritional benefits to large segments of the population in developing countries, although many millions of people in developing countries are still not able to afford high-quality diets [38]. Brazil has an important role in the world milk chain. In 2014, it ranked fifth in the world with respect to milk production, behind the European Union, India, the United States of America, and China. Southeast Brazil, the region most affected by BV, produces about 12.16 billion liters per year, which corresponds to 34.6% of the total national production, milking 34.4% of the total of Brazilian dairy cows [82]. The emergence of BV outbreaks in the country is a public health risk and is responsible for economic losses in the milk production chain [7].

## 5. Concluding Remarks

BV is a zoonotic occupational disease affecting cattle and humans, mainly milk-producing farmers. The finding that BV in cattle is not a localized and self-limited disease brought to light new insights about systemic and prolonged infections. The observation that VACV is shed through feces and milk, even after the complete healing of lesions and from animals with subclinical infection, provides important information related to VACV pathogenesis, transmission, and the virus maintenance cycle in nature. In addition, these data highlight new concerns about the role of VACV in public health. Currently, there is no public health policy regarding BV disease in Brazil. There are no available treatments and no vaccination programs for humans or cattle in Brazil. The pathogenesis model of the disease in cattle provides a better understanding of the immunological response and may facilitate the development of potential tools for the control and prevention of BV. However, it is important to emphasize that although many gaps have been filled, other unknown aspects have appeared, bringing new challenges in the understanding of this disease.
